# Autophagy resolves early retinal inflammation in *Igf1*-deficient mice

**DOI:** 10.1242/dmm.026344

**Published:** 2016-09-01

**Authors:** Ana I. Arroba, Lourdes Rodríguez-de la Rosa, Silvia Murillo-Cuesta, Laura Vaquero-Villanueva, Juan M. Hurlé, Isabel Varela-Nieto, Ángela M. Valverde

**Affiliations:** 1 Alberto Sols Biomedical Research Institute (IIBm) (CSIC/UAM), 28029, Madrid, Spain; 2 Spanish Biomedical Research Centre in Diabetes and Associated Metabolic Disorders (CIBERdem), ISCIII, 28029, Madrid, Spain; 3 Biomedical Research Networking Centre on Rare Diseases (CIBERER), ISCIII, 28029, Madrid, Spain; 4 IdiPAZ Institute for Health Research, Madrid 28029, Spain; 5 Departamento de Anatomía y Biología Celular and IDIVAL, Universidad de Cantabria, 39011, Santander, Spain

**Keywords:** Autophagy, IGF-1, Neurodegeneration, Neuroinflammation, Retina

## Abstract

Insulin-like growth factor-1 (IGF-1) is a growth factor with differentiating, anti-apoptotic and metabolic functions in the periphery, and anti-inflammatory properties in the nervous system. Mice that have mutations in the *Igf1* gene, rendering the gene product inactive (*Igf1^−/−^*), present with age-related visual loss accompanied by structural alterations in the first synapses of the retinal pathway. Recent advances have revealed a crucial role of autophagy in immunity and inflammation. Keeping in mind this close relationship, we aimed to decipher these processes in the context of the defects that occur during ageing in the retina of *Igf1^−/−^* mice. *Tnfa* and *Il1b* mRNAs, and phosphorylation of JNK and p38 MAPK were elevated in the retinas of 6- and 12-month old *Igf1^−/−^* mice compared to those in age-matched *Igf1^+/+^* controls. In 6-month-old *Igf1^−/−^* retinas, increased mRNA levels of the autophagy mediators *Becn1*, *Atg9*, *Atg5* and *Atg4*, decreased p62 (also known as SQSTM1) protein expression together with an increased LC3-II:LC3-I ratio reflected active autophagic flux. However, in retinas from 12-month-old *Igf1^−/−^* mice, *Nlrp3* mRNA, processing of the IL1β pro-form and immunostaining of active caspase-1 were elevated compared to those in age-matched *Igf1^+/+^* controls, suggesting activation of the inflammasome. This effect concurred with accumulation of autophagosomes and decreased autophagic flux in the retina. Microglia localization and status of activation in the retinas of 12-month-old *Igf1^+/+^* and *Igf1^−/−^* mice, analyzed by immunostaining of Cd11b and Iba-1, showed a specific distribution pattern in the outer plexiform layer (OPL), inner plexiform layer (IPL) and inner nuclear layer (INL), and revealed an increased number of activated microglia cells in the retina of 12-month-old blind *Igf1^−/−^* mice. Moreover, reactive gliosis was exclusively detected in the retinas from 12-month-old blind *Igf1^−/−^* mice. In conclusion, this study provides new evidence in a mouse model of IGF-1 deficiency that autophagy is an adaptive response that might confer protection against persistent inflammation in the retina during ageing.

## Abstract (precis)

**Summary:** *Igf1*-deficient mice show chronic inflammation in the retina, and we reveal that controlling inflammation through autophagy in young mice could prevent loss of retinal function.

## INTRODUCTION

Insulin-like growth factor-1 (IGF-1) is a growth factor with differentiating, anti-apoptotic and metabolic functions in the periphery (Shaw and Grant, 2004; Laviola et al., 2007). IGF-1 is synthesized and secreted by the liver in response to increased concentrations of growth hormone (GH). Concentrations of circulating IGF-1 vary at different developmental stages, increasing during periods of growth such as puberty and decreasing with ageing (Clark et al., 1998; Lacau-Mengido et al., 2000). IGF-1 actions are mediated by the IGF-1 receptor (IGF1R), which belongs to the tyrosine kinase membrane receptor family, of which the insulin receptor is the prototype (Werner and LeRoith, 2014). In the nervous system, IGF-1 has a role as a neurotrophic peptide, triggering pro-survival signalling that activates anti-apoptotic cascades, enhances nerve growth and promotes synaptic plasticity. In addition, anti-inflammatory properties have been attributed to IGF-1 (Hu et al., 2015). All these functions are essential for protecting nerve cells against neurodegenerative processes (Varela-Nieto et al., 2013; Yamamoto et al., 2014). In fact, IGF-1 resistance in the central nervous system (CNS) impairs neuroprotection in Alzheimer (Steen et al., 2005) and Parkinson diseases (Trejo et al., 2004). Individuals with homozygous mutations of the *IGF1* gene exhibit microcephaly, mental retardation and bilateral sensorineural deafness (Woods et al., 1996; Walenkamp et al., 2005; Walenkamp and Wit, 2007). The therapeutic potential of IGF-1 has been demonstrated in animal models of a number of neurodegenerative diseases such as cerebellar ataxia (Fernandez et al., 2005), multiple sclerosis (Chesik et al., 2007) and, as mentioned above, Parkinson disease (Ebert et al., 2008) and Alzheimer disease (Fernandez and Torres-Aleman, 2012), in which treatment with IGF-1 alleviates neurological symptoms.

Age-related loss of sensory activity represents a costly and socially debilitating aspect in general senescence of the CNS. Our previous studies in mice that lacked *Igf1* have demonstrated that these mice present congenital sensorineural deafness and age-related metabolic cochlear alterations (Camarero et al., 2001; Cediel et al., 2006; Riquelme et al., 2010; Sanchez-Calderon et al., 2010). Moreover, mice deficient in IRS2 (*Irs2*^−/−^), a downstream docking molecule of the IGF1R signaling cascade, also exhibit congenital sensorineural deafness, which can be detected before the onset of diabetes, that is characterized by altered cochlear morphology with hypoinnervation of the cochlear ganglion and aberrant stria vascularis (Murillo-Cuesta et al., 2012). Interestingly, some of the alterations in the gene expression profile found in *Igf1^−/−^* mice are related to retinal development (Sanchez-Calderon et al., 2010). These results were confirmed by analysis of visual function, which revealed a loss of vision over time in *Igf1^−/−^* mice with a very small amplitude in the electroretinogram (ERG) waves at the age of 12 months (Rodriguez-de la Rosa et al., 2012). Importantly, the defect in visual function is accompanied by a significant loss of cell contacts in the outer plexiform layer (OPL) between the photoreceptors and their postsynaptic bipolar and horizontal cells. However, the molecular events that compromise retinal structure and visual function in *Igf1^−/−^* mice during ageing have not been investigated in depth.

During chronic diseases of the retina, a close association between neurodegeneration and neuroinflammation has been reported. In the CNS, microglial cells comprise the resident phagocyte population, and have important roles in immune surveillance as well as in neuronal homeostasis (Hanisch, 2002; Streit, 2002, 2005). Activated microglia can exert both protective and deleterious functions. In the early phase of neurodegeneration, microglia participate in tissue remodelling and initiate repair mechanisms, such as those during glial scar formation. However, in the CNS, excessive or prolonged activation of microglia, particularly in the retina, leads to chronic inflammation with severe pathological side effects, often resulting in irreversible retinal degeneration (Graeber and Streit, 2010). Interestingly, age-related disorders in the CNS have been attributed to chronic neuroinflammation that results from persistent activation of microglia (Caldeira et al., 2014).

Among the potential biological processes that alleviate neuroinflammation-induced cellular damage, autophagy plays a key role because it is a catabolic process that sequesters components of the cytoplasm, including aberrant organelles and macromolecules, into double-membraned vesicles and delivers them to lysosomes for degradation, leading to eventual recycling of the resulting macromolecules (Klionsky et al., 2016). Autophagy influences the physiology and pathology of many immune cells, including those of microglia. For instance, it has been recently reported that autophagy in microglia degrades extracellular β-amyloid fibrils and regulates the NLRP3 inflammasome (Cho et al., 2014). Moreover, activation of the autophagy process mitigates the expression of proinflammatory cytokines and the cell death of BV2 mouse microglial cells that have been challenged with bacterial lipopolysaccharide (Han et al., 2013). Keeping in mind this close relationship between neuroinflammation and autophagy, our aim in this study was to decipher these processes in the context of the retinal defects that occur during ageing in *Igf1^−/−^* mice.

## RESULTS

### Igf1 deficiency increases retinal inflammatory markers

**Fig. 1.**
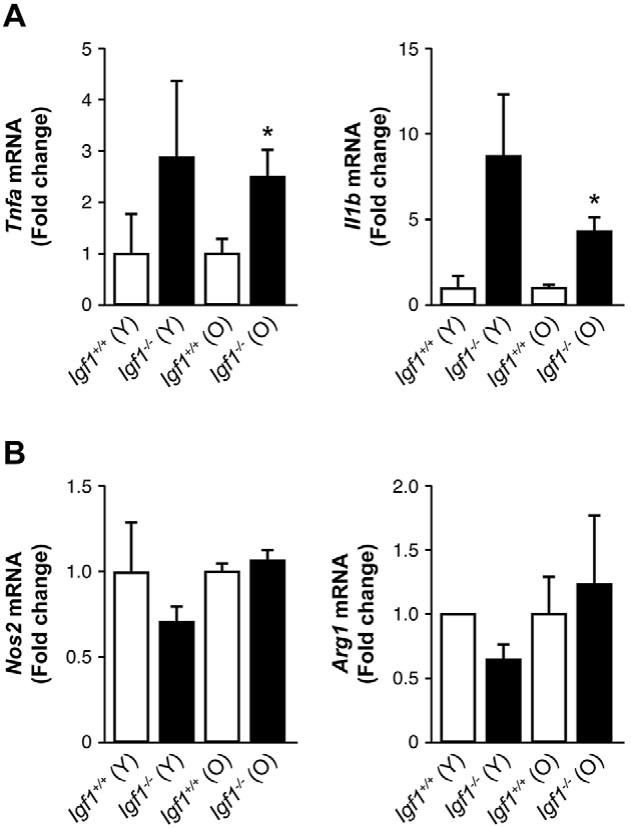
**Analysis of proinflammatory markers in the retinas from *Igf1*^−/−^ mice during ageing.** *Tnfa*, *Il1b* (panel A), *Nos2* and *Arg1* (panel B) mRNA levels in young (Y) and old (O) *Igf1^−/−^* and *Igf1*^+/+^ mice matched at each age. Results are means±s.e.m. (*n*=5 retinas per experimental condition). The fold change relative to *Igf1^+/+^* (Y) is shown. *P≤0.05, *Igf1^−/−^* vs *Igf1*^+/+^ mice matched at each age (two-way ANOVA followed by Bonferroni *t*-test).

Based on our previous study in wild-type (*Igf1^+/+^*) and *Igf1^−/−^* mice (Rodriguez-de la Rosa et al., 2012), we first evaluated inflammation in the retina in those mice at two distant ages; 6 months (referred to in figures as young, Y) with unaltered visual function and 12 months (referred to in figures as old, O) at which there was profound loss of vision. As shown in Fig. 1A, elevations in *Tnfa* and *Il1b* mRNAs were found in the retinas of *Igf1^−/−^* mice at both ages compared to the values of their respective age-matched wild-type (*Igf1^+/+^*) counterparts. Of note, mRNA levels of *Il6* were not detected in the retinas of both genotypes of mice (results not shown). Moreover, we analyzed mRNA levels of *Nos2* as a marker of the M1 macrophage proinflammatory response and *Arg1* mRNA (encoding arginase-1), a marker of the M2 macrophage anti-inflammatory response, and no differences among groups were found (Fig. 1B).

**Fig. 2.**
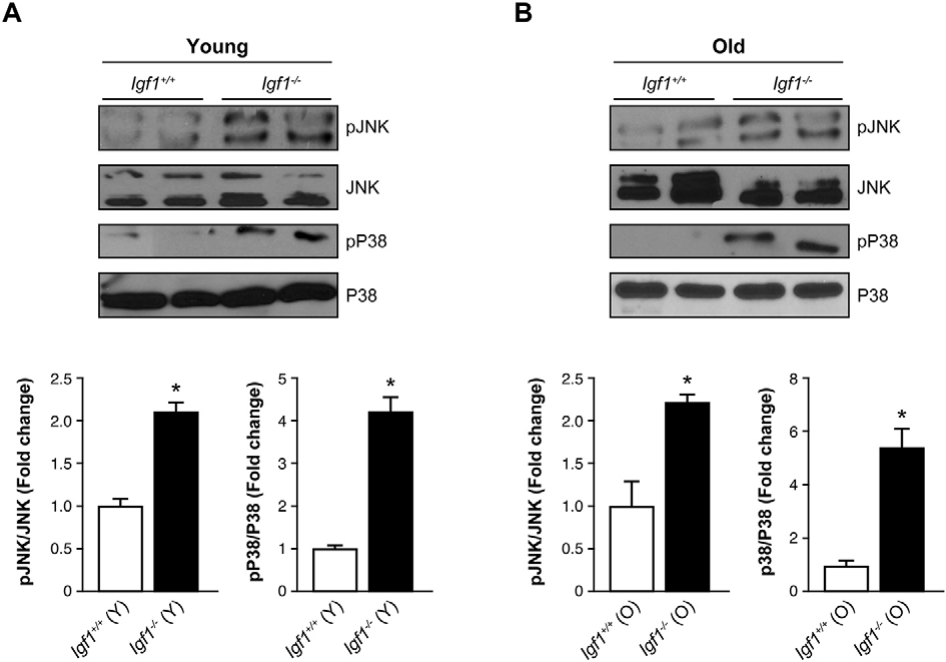
**Activation of stress-kinase-mediated signaling in the retinas of *Igf1*^−/−^ mice.** Protein extracts (30 μg) from retinas from young (Y) and old (O) (panels A and B, respectively) *Igf1^−/−^* and *Igf1*^+/+^ mice were separated by SDS-PAGE and analyzed by western blotting with antibodies against phosphorylated (p)-JNK, total JNK, phosphorylated (p)-p38 MAPK and total p38 MAPK. Two samples from different mice are shown for each genotype and age. Representative autoradiograms are shown (*n*=6 retinas per experimental condition). Blots were quantified by performing scanning densitometry, and results are means±s.e.m. *P≤0.05, *Igf1^−/−^* vs *Igf1*^+/+^ mice matched at each age (two-way ANOVA followed by Bonferroni *t*-test). / indicates the ratio between the two indicated proteins, and the fold change relative to *Igf1*^+/+^ mice is shown.

We also evaluated the impact of *Igf1* deficiency in the activation of proinflammatory signaling pathways in the retina. As Fig. 2A,B shows, increased phosphorylation of JNK and p38 MAPK was found in young and old *Igf1^−/−^* mice compared to their age-matched *Igf1^+/+^* counterparts, reflecting chronic inflammation in these mice at both ages. Two-way ANOVA analysis revealed significant differences among genotypes (*Igf1^−/−^* vs *Igf1^+/+^*) at each age, but not when comparing the responses at the two different ages (old vs young) within each genotype.

### Autophagy flux is activated in the retina from young *Igf1^−/−^* mice

**Fig. 3.**
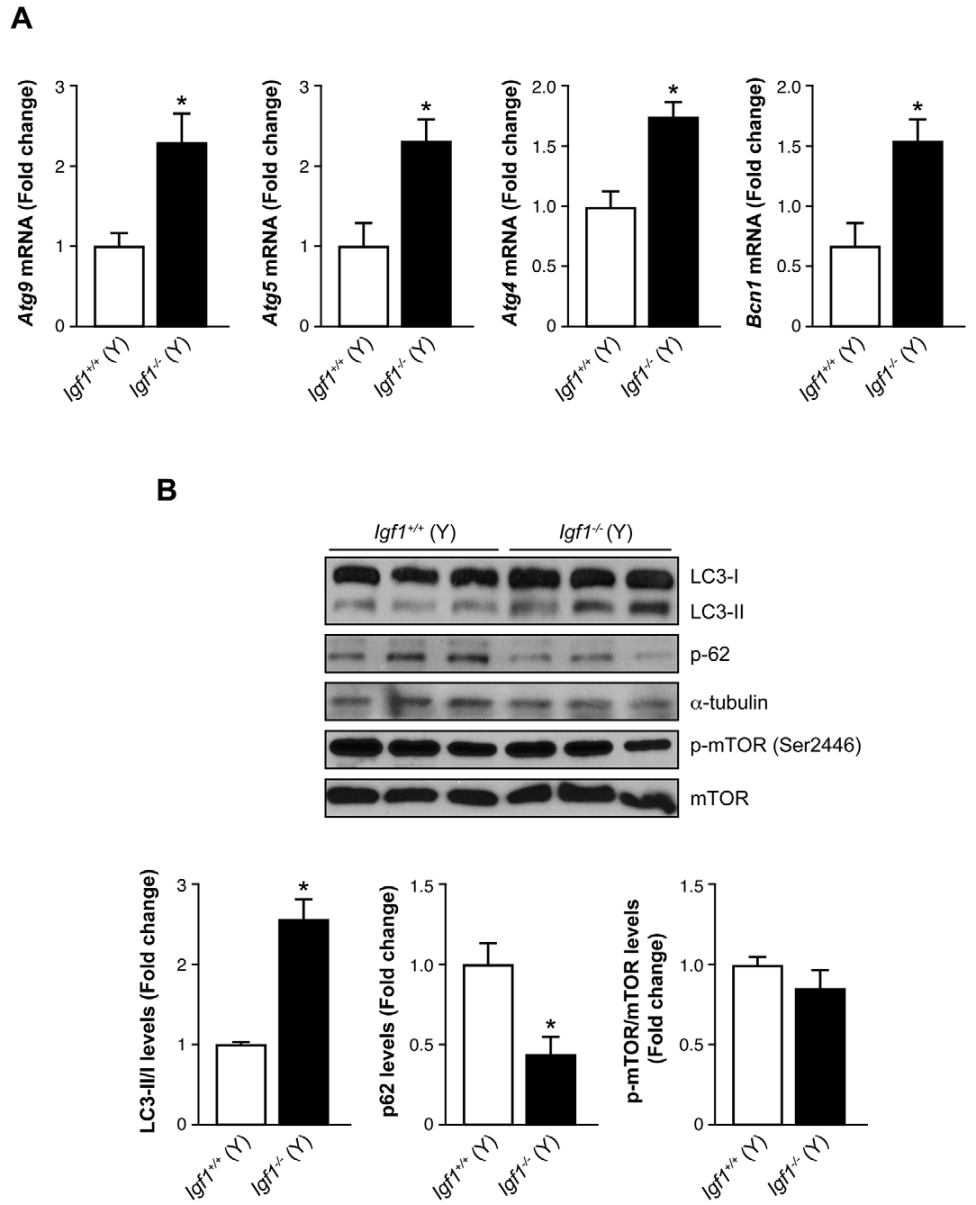
**Analysis of the autophagic flux in retinas from young *Igf1*^−/−^ mice.** (A) *Atg9*, *Atg5*, *Atg 4* and *Bcn1* mRNA levels in retinas from young (Y) *Igf1^−/−^* and *Igf1*^+/+^ mice (*n*=6 retinas per genotype). (B) Protein extracts (30 μg) from retinas from young (Y) *Igf1^−/−^* and *Igf1*^+/+^ mice were separated by SDS-PAGE and analyzed by western blotting with antibodies against LC3, p62 and phosphorylated mTOR. α-Tubulin was used as a loading control. Three samples from different mice are shown for each genotype and age. Representative autoradiograms are shown (*n*=6 retinas per genotype). The blots were quantified by performing scanning densitometry, and results are means±s.e.m. *P≤0.05, *Igf1^−/−^* vs *Igf1*^+/+^ mice (two-way ANOVA followed by Bonferroni *t*-test). / indicates the ratio between the two indicated proteins, and the fold change relative to *Igf1*^+/+^ (Y) is shown.

Recently, autophagy has emerged as a biological process related to inflammation in different cellular contexts because it targets the inflammasome components for degradation (Harris, 2011; Shi et al., 2015). On that basis, we hypothesized that differences in autophagy could explain the differences between young and old *Igf1^−/−^* mice in the retinal structure and visual function that have already been detected (Rodriguez-de la Rosa et al., 2012). The mRNA levels of autophagy mediators, such as *Becn1*, *Atg9*, *Atg5* and *Atg4*, were significantly elevated in young *Igf1^−/−^* retinas compared to in the age-matched *Igf1^+/+^* controls (Fig. 3A). As lipidation of LC3 is a useful marker for autophagy, we analyzed LC3 by western blotting, and we detected a significant increase in the LC3-II:LC3-I ratio (2.3-fold) in the retinas from young *Igf1^−/−^* mice as compared with that in age-matched *Igf1^+/+^* controls (Fig. 3B). Increased LC3-II indicates an accumulation of autophagosomes, which does not always mean the upregulation of the autophagic flux (Klionsky et al., 2016). Thus, autophagic function was evaluated by analysing protein levels of p62 (also known as SQSTM1), a selective substrate of autophagy, because activation of the autophagic flux is associated with a decline in p62 protein levels, and vice versa (Bjorkoy et al., 2005). As Fig. 3B shows, a significant decrease in p62 was detected in young *Igf1^−/−^* retinas, reflecting an active autophagic flux at this age. To inspect the signaling pathways involved in autophagy, we evaluated the activation of mammalian target or rapamycin (mTOR) as a well-established signaling mediator of autophagy. The data in Fig. 3B show that there were no differences in mTOR phosphorylation levels in the retina of the two genotypes of mice.

### Analysis of the inflammasome in the retina of *Igf1*^−/−^ mice during ageing

To elucidate the involvement of the inflammasome system in retinal inflammation in *Igf1*^−/−^ mice, we analyzed the expression of caspase-1, a key component of the inflammasome that is responsible for IL1β processing. Neither active caspase-1 (Fig. S1A) nor processing of IL1β (results not shown) were detected in the retinas of young *Igf1^−/−^* mice. In agreement with this, no differences were found in the mRNA levels of *Nlrp3* in the retinas from young *Igf1^+/+^* and *Igf1^−/−^* mice (Fig. S1B).

Next, we evaluated the activation status of microglia and their localization in the retina by performing double immunofluorescence analysis for Cd11b and Iba-1. Specific immunolabelling for the microglia markers Iba-1 and Cd11b was detected in the retinas from young *Igf1^+/+^* and *Igf1^−/−^* mice, and the quantification of the number of immunolabelled cells was similar in both experimental groups (Fig. S1C). Likewise, we did not find differences in Iba-1 protein expression between young *Igf1^+/+^* and *Igf1^−/−^* mice (results not shown).

**Fig. 4.**
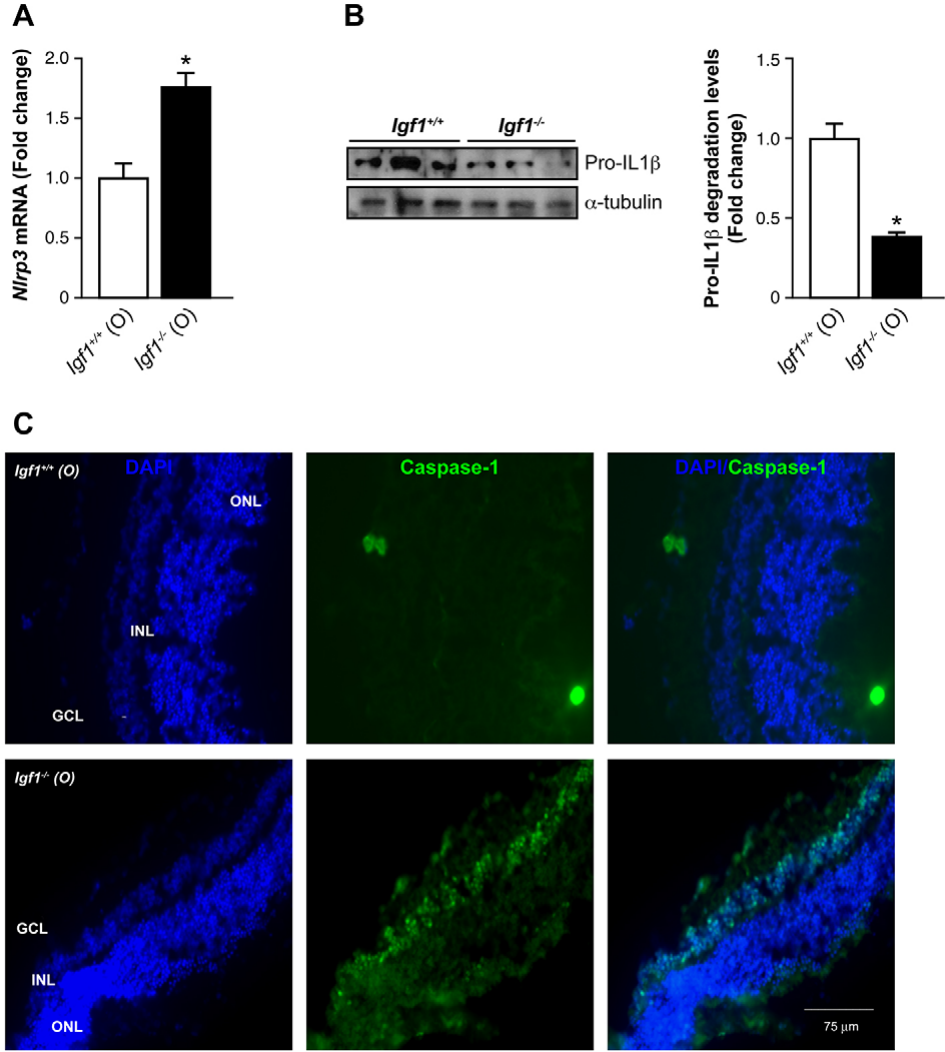
**Analysis of the inflammasome complex in retinas from old *Igf1*^−/−^ mice.** (A) *Nlrp3* mRNA levels in retinas from old (O) *Igf1^−/−^* and *Igf1*^+/+^ mice. Results are means±s.e.m. (*n*=6 retinas per genotype). (B) Protein extracts (30 μg) of retinas from old (O) *Igf1^−/−^* and *Igf1*^+/+^ mice were separated by SDS-PAGE and analyzed by western blotting with an antibody against IL1β. α-Tubulin was used as a loading control. Three samples from different mice are shown for each genotype and age. Representative autoradiograms are shown (*n*=6 retinas per genotype). The blots were quantified by performing scanning densitometry, and results are means±s.e.m. *P≤0.05, *Igf1^−/−^* vs *Igf1*^+/+^ mice (two-way ANOVA followed by Bonferroni *t*-test). Fold changes relative to *Igf1*^+/+^ (O) mice are shown. (C) Immunostaining for caspase-1 (fragment p10; green), and cells were counterstained with DAPI (blue). Representative images are shown. GCL, ganglion cell layer; INL, inner nuclear layer; ONL, outer nuclear layer.

As found in young mice (Fig. S1B), the analysis of the inflammasome system in the retinas of old mice that lacked *Igf1* revealed increased mRNA levels of *Nlrp3* compared to those in their respective age-matched *Igf1^+/+^* controls (Fig. 4A). Two-way ANOVA analysis revealed significant differences in *Nlrp3* mRNA levels across genotypes (*Igf1^−/−^* vs *Igf1^+/+^*) in both young (Fig. S1B) and old (Fig. 4A) mice, but not in the responses compared between the two ages (old vs young) within each genotype (data not shown). This elevation correlated with the processing of the IL1β pro-form given that its levels were significantly decreased in *Igf1^−/−^* mice (Fig. 4B); however, we were unable to detect the IL1β active fragment in retinal extracts. As expected, the immunofluorescence analysis of retinal sections in old *Igf1^−/−^* mice showed higher levels of active caspase-1, mainly localized in the INL, compared to age-matched *Igf1^+/+^* controls (Fig. 4C), indicating the activation of the inflammasome. These results indicate that in old *Igf1^−/−^* mice, neuroinflammation in the retina concurs with activation of the inflammasome and the loss of visual function.

**Fig. 5.**
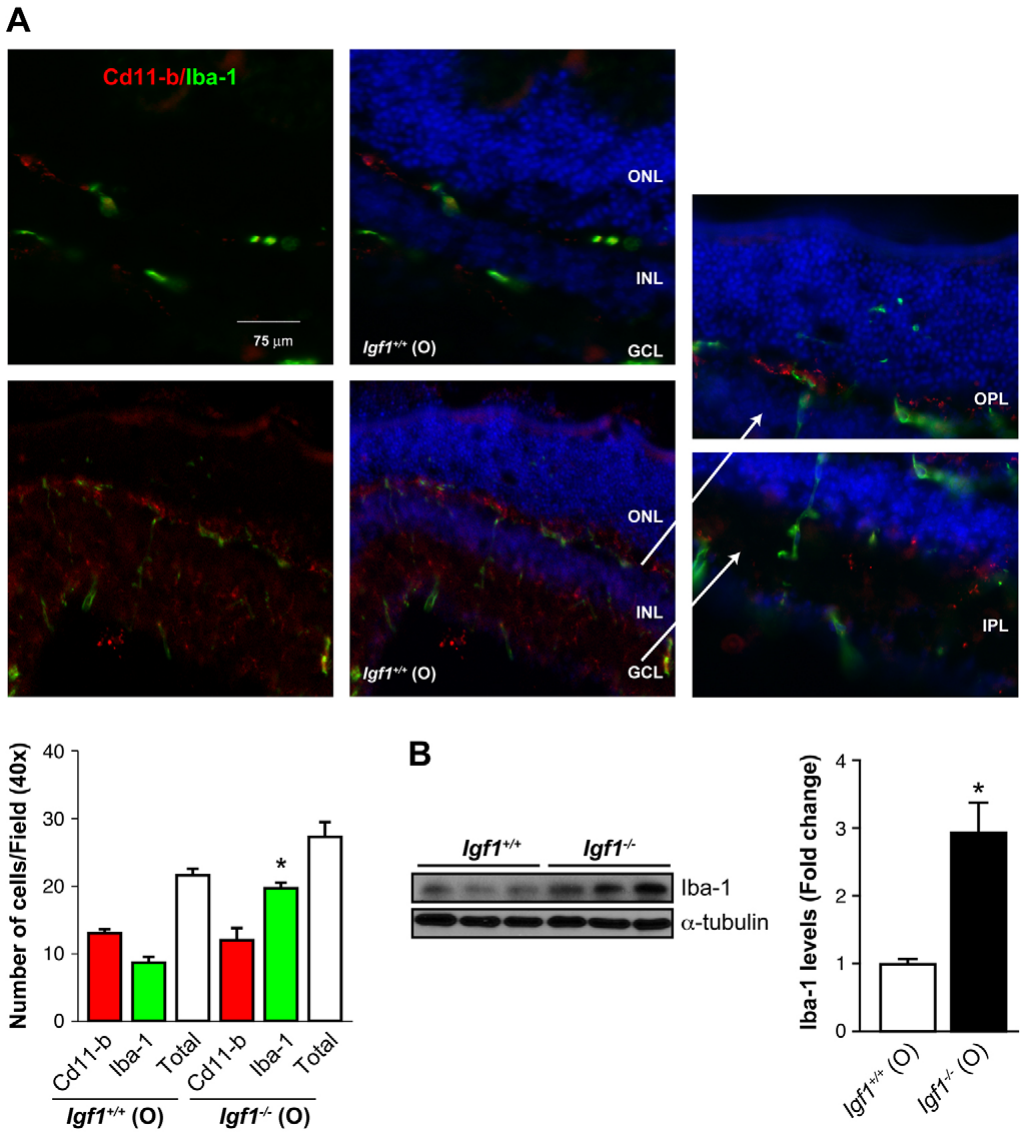
**Increased number of activated microglia cells in retinas from old *Igf1*^−/−^ mice.** Immunostaining (A) and quantification of Cd11b- (red) and Iba-1- (green) positive cells counterstained with DAPI (blue) in retinas from old (O) *Igf1^−/−^* and *Igf1*^+/+^ mice. GCL, ganglion cell layer. The results are means±s.e.m. *P≤0.05, *Igf1^−/−^* vs *Igf1*^+/+^ mice (two-way ANOVA followed by Bonferroni *t*-test). Arrows point to enlarged images of the indicated areas. (B) Protein extracts (30 μg) of retinas from old (O) *Igf1^−/−^* and *Igf1*^+/+^ mice were separated by SDS-PAGE and analyzed by western blotting with the antibody against Iba-1. α-Tubulin was used as a loading control. Three samples from different mice are shown for each genotype and age. Representative autoradiograms are shown (*n*=6 retinas per genotype). The blots were quantified by performing scanning densitometry, and the results are means±s.e.m. *P≤0.05, *Igf1^−/−^* vs *Igf1*^+/+^ mice (two-way ANOVA followed by Bonferroni *t*-test). Fold changes relative to *Igf1*^+/+^ (O) are shown.

Because a switch in the activation status of microglia has been described in experimentally-induced stress and in animal models of neurodegenerative diseases (Streit, 2005), we next evaluated microglia localization and the status of activation in the retinas of old *Igf1^+/+^* and *Igf1^−/−^* mice by performing double Cd11b and Iba-1 immunostaining. Cd11b preferentially labels ramified resting microglia (both recruited and local), whereas Iba-1 preferentially labels amoeboid-activated microglia. The quantification of Iba-1 immunopositive microglial cells revealed a significant increase of that in the retinas from old *Igf1^−/−^* mice as compared with age-matched *Igf1^+/+^* controls, which exhibited a specific distribution in the outer plexiform layer (OPL), inner plexiform layer (IPL) and inner nuclear layer (INL) (Fig. 5A). This result was confirmed by western blot analysis of Iba-1 protein expression in retinal extracts (Fig. 5B). Two-way ANOVA analysis of the number of Iba-1 positive microglial cells in young (Fig. S1C) and old (Fig. 5A) mice revealed significant differences between the genotypes (*Igf1^−/−^* vs *Igf1^+/+^*) only in old mice (data not shown). When comparisons were made between ages (old vs young), significant differences were found within each genotype (data not shown).

### Autophagy declines with ageing in the retina of *Igf1*^−/−^ mice

**Fig. 6.**
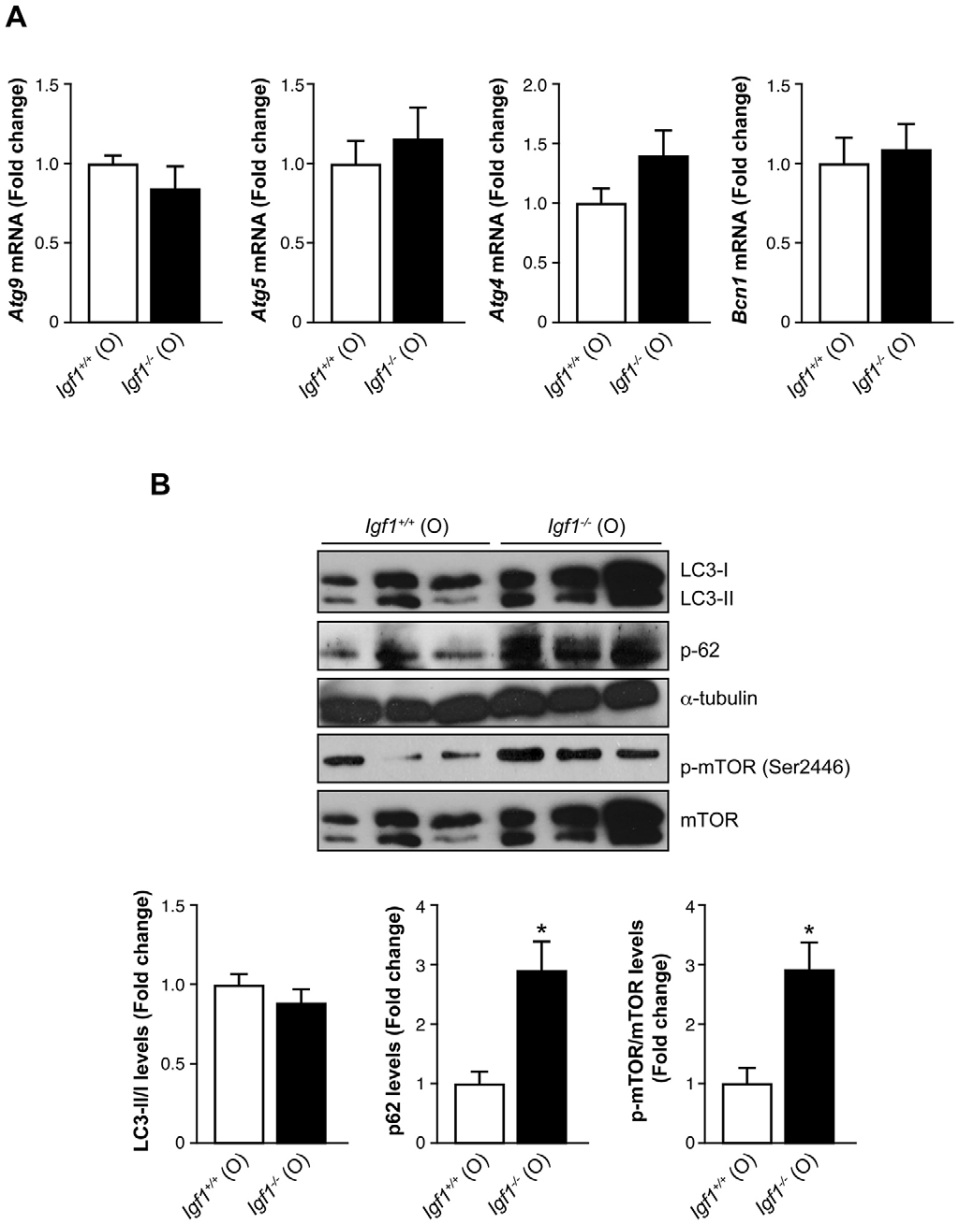
**Decreased autophagy flux in the retina from old *Igf1*^−/−^ mice.** (A) *Atg9*, *Atg5*, *Atg4* and *Bcn*1 mRNA levels in retinas from old (O) *Igf1^−/−^* and *Igf1*^+/+^ mice (*n*=5 retinas per genotype). (B) Protein extracts (30 μg) of retinas from old (O) *Igf1^−/−^* and *Igf1*^+/+^ mice were separated by SDS-PAGE and analyzed by western blotting with antibodies against LC3, p62, phosphorylated (p)-mTOR and mTOR. α-Tubulin was used as a loading control. Three samples from different mice are shown for each genotype and age. Representative autoradiograms are shown (*n*=6 retinas per genotype). The blots were quantified by performing scanning densitometry. The results of panels A and B are means±s.e.m. *P≤0.05, *Igf1^−/−^* vs *Igf1*^+/+^ age-matched mice (two-way ANOVA followed by Bonferroni *t*-test). / indicates the ratio between the two indicated proteins, and the fold change relative to *Igf1*^+/+^ (O) mice is shown.

Opposite to our observations in young mice (Fig. 3), mRNA levels of *Becn1*, *Atg9*, *Atg5* and *Atg4* were similar in old *Igf1^+/+^* and *Igf1^−/−^* mice (Fig. 6A). Two-way ANOVA analysis revealed all these autophagy-related genes significantly decreased with ageing exclusively in *Igf1^−/−^* mice. At this age, retinas from old *Igf1^−/−^* mice showed higher p62 protein levels than those of the *Igf1^+/+^* controls, whereas similar LC3-II:LC3-I ratios were found in the retinas of both genotypes (Fig. 6B), indicating a decrease of the autophagic flux in retinas that lacked *Igf1*. Moreover, increased levels of phosphorylation of mTOR reinforced the blockade of autophagy in retinas from old *Igf1^−/−^* mice. Interestingly, two-way ANOVA analysis showed significant differences in these parameters when comparisons were made between the two ages (old vs young; data not shown) and between genotypes (*Igf1^−/−^* vs *Igf1^+/+^*).

### Ultrastructural features of altered autophagy in the retinas of *Igf1*^−/−^ mice

**Fig. 7.**
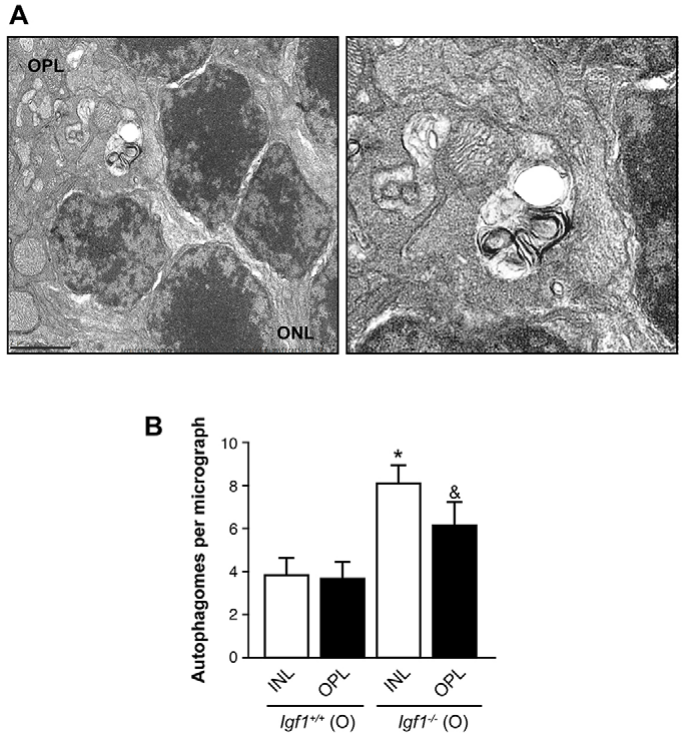
**Accumulation of autophagosomes in retinas from old *Igf1*^−/−^ mice.** (A) In these representative TEM images, typical autophagosomes can be identified by the presence of double membranes in retinas from old (O) *Igf1^−/−^* and *Igf1*^+/+^ mice. Scale bar: 2 µm. (B) The autophagosomes were counted in ten fields per mouse (*n*=3 mice per genotype). The results are means±s.e.m. *P≤0.05, *Igf1^−/−^* vs *Igf1*^+/+^ age-matched mice at the inner nuclear layer (INL). ^&^*P*≤0.05, *Igf1*^−/−^ vs *Igf1*^+/+^ age-matched mice at the outer plexiform layer (OPL). ONL, outer nuclear layer.

To further assess the alteration of the autophagic activity during ageing in the retina of *Igf1*^−/−^ mice, we examined retinal sections with transmission electron microscopy (TEM). We found in the retina of both mouse genotypes double- and multiple-membraned vacuoles containing electron-dense materials or whorls of membranous material, which are properties of autophagosomes (Klionsky et al., 2016) (Fig. 7A). We focused on the analysis of the number of autophagosomes located in the INL and OPL – layers in which inflammatory processes have been detected. Autophagosomes were seen in the cytoplasm of the cells from the INL and were scattered in the dendrites of those cells in the OPL. The average number of autophagosomes in the cytoplasm of cells located at the INL was 3.83/50 mm^2^ in *Igf1*^+/+^ mice compared to 8.12/50 mm^2^ in *Igf1*^−/−^ mice, and the average number of autophagosomes in the OPL was 3.66/50 mm^2^ in *Igf1*^+/+^ mice in comparison to 6.16/50 mm^2^ in *Igf1*^−/−^ mice (Fig. 7B).

### Reactive gliosis in *Igf1*^−/−^ mice occurs during ageing

**Fig. 8.**
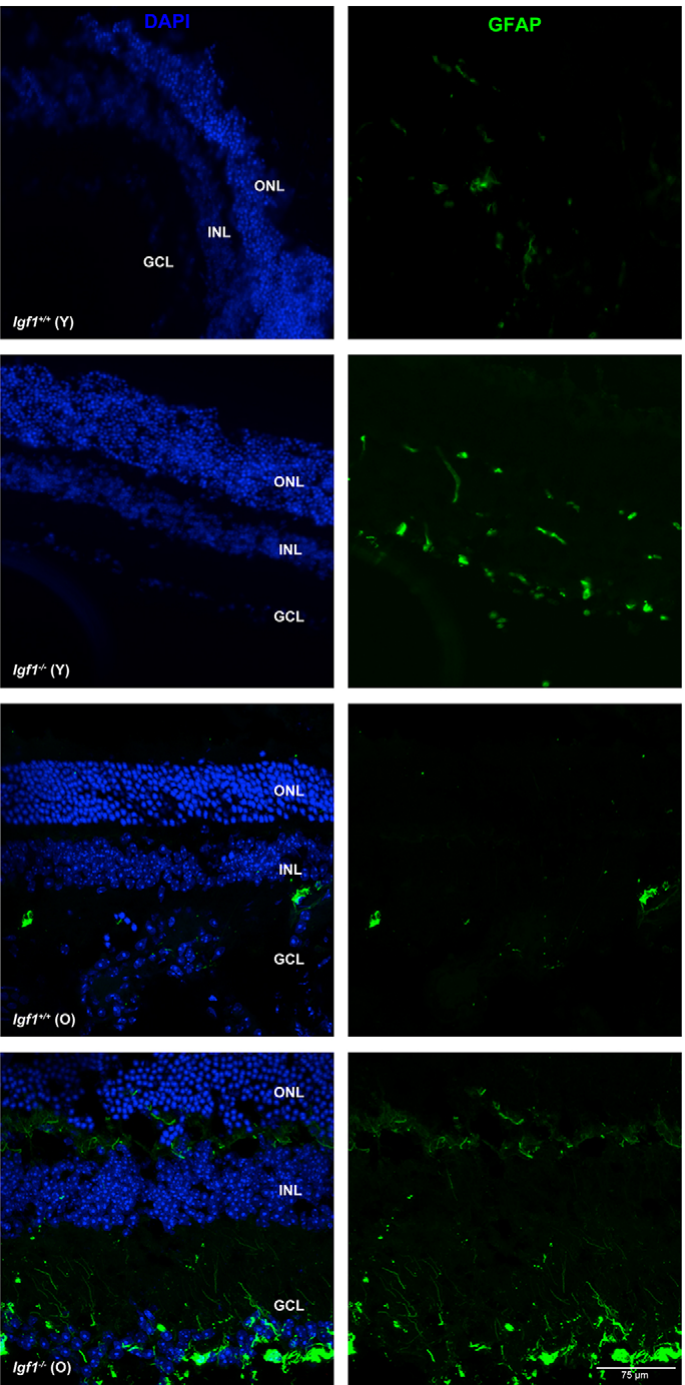
**Reactive gliosis is increased in the retinas from old *Igf1*^−/−^ mice.** Retinal sections from young (Y) and old (O) *Igf1^−/−^* and *Igf1*^+/+^ mice were immunolabelled for GFAP (green) and counterstained with DAPI (blue). Representative images are shown. Merged images are shown on the left, GFAP-only images are shown on the right.

In an attempt to correlate the retinal and visual alterations of *Igf1^−/−^* mice during ageing with persistent unresolved neuroinflammation, we evaluated the glial activation in the retina. Reactive gliosis in macroglia was monitored by the expression of glial fibrillar acidic protein (GFAP). As depicted in Fig. 8, strong GFAP immunostaining was detected in sections of retinas from old *Igf1^−/−^* mice, particularly between the IPL and OPL, whereas the signal was very weak in retinas from *Igf1^+/+^* mice at the same age.

## DISCUSSION

IGF-1 is a neurotrophic and anti-apoptotic factor in the CNS (Guan, 2008). Therefore, the research on IGF-1 actions during neuronal injuries is of great relevance because it could provide opportunities to develop therapeutic approaches to combat neural diseases. IGF-1 enhances neuronal metabolism (Bondy and Cheng, 2002), modulates neuronal excitability (Carro et al., 2000) and also elicits anti-apoptotic, antioxidant and anti-inflammatory roles (Higashi et al., 2010). Very recently, it has been demonstrated that brain-derived IGF-1 plays a crucial role during postnatal hippocampal neurogenesis (Nieto-Estevez et al., 2016). Deficiency in the *IGF1* gene in humans is associated with neuronal disorders such as microcephaly, mental retardation and bilateral sensorineural deafness (Woods et al., 1996; Walenkamp et al., 2005; Netchine et al., 2009).

We have reported that *Igf1*^−/−^ mice have progressive loss their visual function; these animals being almost blind at 12 months of age. This loss of visual function correlates with structural alterations in the first synapse of the retinal pathway and affects the postsynaptic transduction from horizontal and bipolar cells located between the INL and OPL. However, photoreceptor cell number was unaffected by *Igf1* deletion in this mouse model of syndromic deafblindness (Rodriguez-de la Rosa et al., 2012). Moreover, we have recently demonstrated that the enhancement of IGF1R-mediated signaling through inhibition of protein tyrosine phosphatase 1B conferred protection against inflammation-mediated reactive gliosis in the retina (Arroba and Valverde, 2015). Based on these previous findings, in this study we analyzed the inflammatory context in the retina of *Igf1*^−/−^ mice at two ages – 6 months apart – which revealed marked differences in retinal structure and visual function. Notably, increased mRNA levels of proinflammatory cytokines (TNFα and IL1β) were detected in the retinas from *Igf1*^−/−^ mice at 6 months of age, indicating that an incipient proinflammatory status is established before alterations in both retinal synapses and visual function can be detected. Importantly, inflammation remained elevated in old *Igf1^−/−^* mice, reflecting a persistent proinflammatory environment in their retinas. At the molecular level, inflammation was evidenced by increased JNK and p38 MAPK phosphorylation in the retinas of *Igf1^−/−^* mice at both ages. Based on these results, we hypothesized that the chronic inflammatory status in the retina of *Igf1^−/−^* mice at the age of 6 months might be an initial trigger for the retinal and visual defects detected later on. Taking a step further, the activity of the inflammasome system, monitored by examining the mRNA levels of *Nlrp3*, processing of pro-IL1β and the immunostaining of the active fragment of caspase-1 (p10) in the INL, was found to be elevated exclusively in the retinas of old *Igf1^−/−^* mice. Therefore, in the retinas of young *Igf1^−/−^* mice, protective mechanisms might account for the defence against neuroinflammation.

The relationship between IGF-1 and autophagy is becoming a focus of research in the field of neurodegeneration. In fact, little attention has been previously paid to the role of IGF-1 in promoting cell survival against excitotoxicity, which is always associated with the early stages of injury (Troncoso et al., 2014), through regulation of autophagy. Autophagy is a highly sensitive cellular process induced in response to a wide range of stressful conditions in order to maintain homeostasis. Among them, prolonged exposure of tissues and organs to high concentrations of inflammatory mediators represents a stressful environment and can result in severe damage (Cook et al., 2004). Because abnormal inflammation could disrupt cellular homeostasis, autophagy contributes to dampening of inflammatory responses (Lapaquette et al., 2015). Our results clearly showed that, in mice that lack *Igf1^−/−^*, the autophagic flux, monitored by measuring the mRNA levels of autophagy-related genes and LC3-II and p62 protein levels, was enhanced at 6 months of age, a time at which synaptic connectivity in the retina and visual function are well preserved (Rodriguez-de la Rosa et al., 2012). These data are reinforced by the absence of activation of the inflammasome system in the retina despite the increased mRNA levels of *Tnfa* and *Il1b*. Regarding IL1β, although its mRNA levels were higher in the retinas of young *Igf1^−/−^* mice compared to those in their age-matched wild-type counterparts, the absence of activation of the inflammasome system is likely to prevent IL1β processing (Sutterwala et al., 2006). It is intriguing how *Igf1* deficiency is able to trigger autophagy as a protective mechanism that is aimed at preserving retinal structure and visual function. This response deserves further research in the context of neurodegeneration. Based on that, therapies aimed at increasing the autophagic flux are promising in order to prevent the progression of neurodegenerative diseases, particularly those affecting the retina (Rodriguez-Muela et al., 2015).

The opposite situation was detected in retinas of old *Igf1^−/−^* mice. In those mice, the activation of the inflammasome (increased *Nlrp3* mRNA, immunolabelling of active caspase-1 and IL1β processing) coincided with the blockade of the autophagic flux, which was revealed by elevations in mTOR phosphorylation and p62 levels in whole retinal extracts. Confirmation of these results was obtained with TEM analysis that revealed the accumulation of autophagosomes in the INL and OPL layers of the retina in *Igf1^−/−^* mice at the age of 12 months. Because our previous work demonstrates that, in these layers, neural synapsis is disrupted (Rodriguez-de la Rosa et al., 2012), we can hypothesize that, in neuronal cells of the INL and OPL, autophagy might be necessary to preserve photoreceptor connectivity in the retina (Shen et al., 2015), a process which is likely to be negatively affected by the proinflammatory environment. Interestingly, the accumulation of autophagosomes in these layers is coincident with the presence of activated (amoeboid) microglia. It is well known that in healthy retina, ramified microglia cells (Cd11b^+^) are constantly involved in the surveillance of the retinal layers by releasing neuroprotective and anti-inflammatory factors (Streit, 2002; Arroba et al., 2011; Zabel and Kirsch, 2013). However, in response to a persistent proinflammatory stimuli, the chronically activated amoeboid-shaped microglia can induce neuroinflammation by releasing a plethora of cytokines (Block et al., 2013; Mosher and Wyss-Coray, 2014). Our results have revealed that, in the retina of old *Igf1^−/−^* mice, activated amoeboid microglia (Iba-1^+^) are present to a higher extent than in age-matched wild-type mice, showing a distribution pattern around the IPL, OPL and INL. As stated above, these are the crucial layers where cells that are responsible for the amplification of the synaptic transmission and that are highly sensitive to the inflammatory environment are located. Therefore, the inhibition of the autophagic processes in the retina from old *Igf1*^−/−^ mice could explain the advanced state of retinal gliosis in these mice, which is a hallmark of neurodegeneration (Xing et al., 2012; Arroba et al., 2014).

In conclusion, this study provides new evidence regarding the effect of *Igf1* deficiency on the blockade of autophagy in the retina that is related to neuroinflammation. Our results pinpoint autophagy as an adaptive response that might confer protection against the persistent activation of the inflammasome and microglia in the retina during ageing, therefore protecting against deterioration of the retinal synapses and visual function. In this scenario, malfunction of aged microglia can trigger a chronic low-grade inflammatory environment that favours the onset and progression of retinal degeneration.

## MATERIAL AND METHODS

### Mouse handling and genotyping

Heterozygous mice with a targeted disruption of the *Igf1* gene were bred and maintained on a hybrid MF1 and 129/sv genetic background to increase survival of nullizygous *Igf1* mutants (Liu et al., 1993). The mortality of null mice before adulthood is high, although between 20 and 30% of mice survive after birth. Male and female wild-type (*Igf1^+/+^*) and null (*Igf1^−/−^*) mice were studied at 6 and 12 months of age. The latter was the oldest age studied because *Igf1^−/−^* mice become totally deaf and blind at this age (Rodriguez-de la Rosa et al., 2012). Hearing was assessed by auditory brainstem recording in the mice, as described previously (data not shown) (Cediel et al., 2006). Mouse genotypes were identified as previously described (Sanchez-Calderon et al., 2010). Mice were fed tap water, standard diet and housed following recommendations of Federation of European Laboratory Animal Science Associations. All animal experimentation was conducted in accordance with Spanish and European legislation (EU directive 2010/63/EU) and approved by the Animal Care and Use Committee of Spanish National Research Council [Consejo Superior de Investigaciones Científicas (CSIC)]. The number of retinas used in each experimental approach, obtained from different mice, is indicated in the figure legends.

### Isolation of retina

Animals were killed by cervical dislocation, and eyes were enucleated. The lens, anterior segment, vitreous body, retinal pigment epithelium and sclera were removed, and the retina was immediately frozen at −80°C for protein or RNA extraction later.

### Quantitative real-time PCR analysis

Total RNA was extracted with Trizol^®^ reagent (Invitrogen, Madrid, Spain) and reverse transcribed using a SuperScript™ III First-Strand Synthesis System for quantitative real-time PCR (RT-qPCR) following the manufacturer's indications (Invitrogen). Quantitative PCR was performed with an ABI 7900 sequence detector. Primer-probe sets for mouse *Tnfa*, *Il6*, *Il1b*, *Nlrp3*, *Nos2*, *Arg1*, *Atg9*, *Atg5*, *Atg4*, *Bcn1* and *18s* were purchased as pre-designed TaqMan gene expression assays (Applied Biosystems).

### Western blotting

Whole retinas were homogenized in lysis buffer containing 50 mM Tris-HCl, pH 7.4, 150 mM NaCl, 1 mM Na_3_VO_4_, 1 mM NaF, 1 mM EGTA, 15% (w/v) NP40 and 0.25% (w/v) sodium deoxycholate, supplemented with protease inhibitors (10 µg/ml leupeptin, 10 µg/ml aprotinin and 100 µg/ml phenylmethylsulphonyl fluoride). All debris was removed by centrifugation at 14,000 ***g*** for 10 min at 4°C, and protein concentration was quantified using the Bio-Rad protein assay with BSA as a standard. Equivalent amounts of protein were resolved using denaturing sodium dodecyl sulphate-polyacrylamide gel electrophoresis (SDS-PAGE), followed by transfer to PVDF membranes (Bio-Rad). Membranes were blocked using 5% non-fat dried milk or 3% BSA in 10 mM Tris-HCl, 150 mM NaCl, pH 7.5 (TBS), and incubated overnight with several antibodies (1:2000 unless otherwise stated) in 0.05% Tween-20 in TBS. Immunoreactive bands were visualized using the enhanced chemiluminescence reagent (Bio-Rad). Primary antibodies against caspase-1 (p10) (#sc-514), iNOS (sc-650), JNK (#sc-571), phosphorylated p38 MAPK (at residues Thr180 and Tyr182) (sc-17852-R) and p38 MAPK (sc-9212) were purchased from Santa Cruz Biotechnology. Anti-phosphorylated-JNK (at Thr183 and Tyr185) (#4668), p62 (#5114) and LC3 (#2775) antibodies were purchased from Cell Signaling Technology. Anti-Iba-1 (#ab5076) and anti-Cd11b (#ab8878) antibodies were from Abcam. Anti-GFAP antibody (#z0334) was obtained from DAKO and anti-α-tubulin (#T-5168) antibody was from Sigma Chemical Co.

### Immunofluorescence

Eyes were fixed in 4% paraformaldehyde for 24 h at 4°C and infiltrated with sucrose 25% (w/v). For immunofluorescence analysis, retinal cryosections were washed in TBS containing 0.1% (w/v) BSA and 0.1% (v/v) Triton X-100 (this buffer was used for all subsequent washes), and then blocked and permeabilized for 2 h in TBS containing 3% (w/v) BSA and 1% (v/v) Triton X-100. The sections were then incubated overnight in a humid chamber at 4°C with rabbit anti-GFAP (1:250), goat anti-Iba-1 (1:100), rat anti-Cd11b (1:250) and rabbit anti-caspase-1 (1:100) antibodies in blocking solution. Sections were washed and incubated for 90 min with anti-rabbit, anti-goat or anti-rat immunoglobulin antibodies conjugated to Alexa-647 or -488 (1:2000; Molecular Probes). After washing, sections were mounted with medium (Fluoromount G) containing 4-6-diamidino-2-phenylindole (DAPI). Staining was observed with an inverted laser confocal microscope LSM710 (Carl Zeiss Microscopy GmbH).

### Microscopy and cell quantification

Images were acquired by using confocal microscopy, and stained cells or nuclei were scored by two observers that had been blinded to the experiment. Stained nuclei of microglial-positive cells were counted in the indicated retinal layers of an entire section of the retina using a 40× objective. At least three retinas and four nonadjacent sections per retina were scored for each experimental point.

### Transmission electron microscopy

Eyes were dissected, fixed in 2% glutaraldehyde in phosphate or cacodylate buffer, postfixed in osmium tetroxide and embedded in araldite. For light microscopy, semi-thin sections were obtained and stained with Toluidine Blue. For TEM analysis, ultrathin sections were stained with lead citrate and examined with a JEOL1011 electron microscope. Quantification of the double-membraned vacuole-like structure was performed by counting the autophagosomes in 50 micrographs (2500 μm^2^ total). The total number of these structures, overlying at least one intersection of a Photoshop-generated grid, was counted.

### Statistical analysis

Densitometry analysis of the western blots was performed using the ImageJ program. Values in all graphs represent the mean±s.e.m. Statistical tests were performed using SPSS 21.0 for Windows (SPSS Inc. IBM). Data were analyzed by using one-way or two-way ANOVA followed by Bonferroni *t*-test. Differences were considered significant at *P*≤0.05.
